# Pediatric Radiology in Era of COVID-19, International Consensus and What Lies Beyond Pneumonia: A Review

**DOI:** 10.31729/jnma.7122

**Published:** 2021-11-30

**Authors:** Pradeep Raj Regmi, Isha Amatya, Sharma Paudel, Prakash Kayastha

**Affiliations:** 1 Department of Radiology, Hospital for Advanced Medicine and Surgery, Dhumbarahi, Kathmandu, Nepal; 2 Nepal Health Research Council, Kathmandu, Nepal; 3 Department of Radiology, Tribhuvan University Teaching Hospital, Maharajgunj, Kathmandu, Nepal

**Keywords:** *children*, *consensus*, *COVID-19*, *radiology*, *systemic*

## Abstract

Diagnostic radiology plays a crucial role in children. The pediatric population has been less studied than the adult population since the initial period of the COVID-19 pandemic to date. Realizing the potential utility of structured reporting, different guidelines and international consensus statements regarding COVID-19 in the pediatric population have been released in recent times. Different clinical and radiological manifestations in children have been evolving in this period of uncertainty and are different from the adult population in certain aspects. Apart from the involvement of lungs, a multisystemic inflammatory syndrome in children or pediatric multi systemic inflammatory syndrome is unique in children. Therefore, awareness of the recent consensus, structural uniform reporting and multi-organ involving patterns in COVID-19 can guide radiologists for a better understanding of this complex novel disease leading to early diagnosis and timely treatment of affected children.

## INTRODUCTION

Coronavirus belongs to the family Coronaviridae and the order Nicovarales, a family that includes the virus ranging from the common cold to Severe Acute Respiratory Syndrome (SARS) and middle-east respiratory syndrome (MERS).^1,2^ The first adult case of COVID-19 was reported on 31^st^ December 2019 in Wuhan, China. The first recorded case outside China was on 13^th^ January 2020 in Thailand.^3^ World Health Organization (WHO) declared COVID-19 as a pandemic disease on 11^th^ March, 2020.^4^ Globally, there have been 164,409,804 confirmed cases of COVID-19 including 3,409,220 deaths till 20^th^ May 2021.^3^ The first case of COVID-19 in Nepal was recorded on 13^th^ January 2020, in a 32-year-old man traveling from Wuhan to Kathmandu.^5^ As of 20^th^ May, there have been 480,418 confirmed cases of COVID-19 with 5,657 deaths, reported to WHO from Nepal.^3^ Infections by COVID-19 continue to increase worldwide and Nepal is facing the second wave of the pandemic.

The first confirmed case in children was reported in January 2020 in Schenzen, China.^6^ Children comprise 22% of the USA (United States of America) population and according to the Centers for Disease Control and Prevention (CDC), approximately 14% of all cases of COVID-19 were among children.^7,8^ Exact number of children affected with COVID-19 is not available in Nepal. However, 121 children were diagnosed with COVID-19 in the government-based COVID care centers in Kathmandu from January 2020 till August 2020. Fever was the most common symptom in 18.2% of children. Most of the pediatric population are asymptomatic (71.9%) or had the mild disease (22.3%) and 4.9% had moderate to severe symptoms.^9,10^ In the cross-sectional study of 46 North American PICUs, between March 14 and April 3, 2020, 48 children were admitted to 14 PICUs in the US and none in Canada. A total of 40 children (83%) had pre existing underlying medical conditions, 35 (73%) presented with respiratory symptoms, and 18 (38%) required invasive ventilation, and the hospital mortality rate was 4.2%.^11^ Pediatric population has been less studied is compared to the adult population affected by this mysterious disease.

## PATHOGENESIS AND TRANSMISSION

The large number of people that were exposed to the wet animal market in Wuhan city were infected, most likely of zoonotic origin. Person-to-person transmission occurs primarily via direct contact or through the droplets spread by coughing or sneezing from the affected individual.^2,3^ The major route of transmission is through respiratory droplets. However, the viral RNA samples were detected in samples of gastrointestinal tracts (saliva and rectal) of children which probably indicates the possible other routes of transmission in children.^12-15^ In Nepal, the majority of children (83.4%) were identified as a part of contact tracing, 28.1% had an identified contact to a person with COVID-19 before their diagnosis and 20.7% had another household member diagnosed with COVID-19.^9^ Other manifestation of COVID-19 in children are severe respiratory infections or multisystem inflammatory disorder (MIS-C) with overlapping features with Kawasaki disease and toxic shock syndrome. Children with more than one comorbid condition have a higher risk of having severe disease. The clinical manifestations in children are milder compared to adults. The exact cause of this difference is unknown. However, there are some hypotheses such as viral load are lower in children than adults and under-expression of the angiotensin-converting enzyme-2, the binding receptor for COVID-19 spike protein.^10-15^

## ROLE OF IMAGING

Diagnostic radiology plays an even more important role in the diagnosis, treatment, monitoring, and follow-up of pediatric patients. Different guidelines and consensus have been published for imaging of children with COVID-19. However, imaging is not indicated for screening purposes or in a child with the mild disease unless a patient has risk factors for progression or develops worsening symptoms.^16^ Mild disease signifies the patient with clinical symptoms such as fever, cough, mild dyspnea, and/or rhinorrhea whereas moderate to severe signifies patient with signs of more serious respiratory compromise (moderate to severe dyspnea or hypoxemia) or symptoms of cardiovascular compromise and/or pending shock (chest pain, tachycardia, hypotension, or altered mentation). In RT-PCR positive patients with co-morbid status like asthma, cystic fibrosis, tuberculosis, HIV, or immune-compromised state, chest imaging is recommended as a baseline and for another possible differential diagnosis. In moderate to severe disease, in resource-limited settings (unavailable of adequate lab facilities and long turnaround time), imaging may be used in the initial phase in presumed positive cases which significantly affects patient management. Serial imaging is advised in moderate to severe disease as needed to access the response to therapy, evaluate clinical deterioration, or assess the positions of life-supporting devices. Post recovery chest imaging is not advised for the asymptomatic mild disease. However, in asymptomatic patients with initial moderate to the severe disease having clinical concerns for long-term lung injury and prior COVID-19 patients with long-term symptoms, follow-up imaging may be done.^17-19^

Chest X-Ray (CXR) is the first go-to modality in cases with moderate or severe disease. According to the American College of Radiology (ACR), imaging is not indicated in screening and in well-appearing immune-competent children more than 3 months old who do not require hospitalization.^18^ However, similar to other viral infections, imaging is considered if the child does not respond to outpatient treatment, who requires hospitalization, and who is suspected of having community-acquired pneumonia. Chest Computed Tomography (CT) can be used to answer the specific question in a child with raised D-dimer and suspicion of pulmonary embolism. Follow-up imaging might be required in a child with the progressive disease with development and evolution of pulmonary fibrosis and significant alteration in pulmonary function test in a child with post-COVID-19 pneumonia. When performing imaging in children, multiple factors like availability of RT-PCR tests, the sensitivity of early imaging, and radiation dose should be taken into consideration.

Recommended structural reporting formats for CXR in children with COVID-19 are^19,20^:

1. **Typical:** Bilateral peripheral/subpleural ground-glass opacities (GGOs)/consolidation with suggested reporting language as imaging findings commonly seen with COVID-19 pneumonia in children. Other differentials include viral or atypical pneumonia.
2. **Indeterminate:** Non-specific findings of COVID-19 pneumonia and includes both infectious and non-infectious etiologies. Unilateral peripheral or peripheral and central GGOs and/or consolidation. Bilateral peribronchial thickening and/or peribronchial opacities. Multifocal or diffuse GGOs and/or consolidation without specific distribution.
3. **Atypical:** Uncommon or not reported imaging findings and recommend consideration of another diagnosis. Unilateral segmental or lobar consolidation, central unilateral or bilateral GGOs and/or consolidation, single round consolidation (round pneumonia with or without air bronchogram), pleural effusion, and lymphadenopathy.
4. **Negative:** No chest radiographic findings suggestive of pneumonia.

Recommended structural reporting formats for chest CT in children with COVID-19 are: ^19,20^

1. **Typical:** Bilateral peripheral and/or subpleural GGOs/consolidation predominant in lower lobes. "Halo sign" in early cases i.e. subtle area of ground-glass opacities with central consolidation which later progresses to frank ground-glass opacities (progressive phase) and consolidation pattern in developed phase. Differential diagnosis includes other viral or atypical pneumonia, hypersensitivity pneumonitis, eosinophilic lung disease, and fungal infection (especially in an immune-compromised child with halo sign).
2. **Indeterminate:** Unilateral peripheral or peripheral/ central GGOs and/or consolidation. Bilateral peribronchial thickening and/or peribronchial opacities. Multifocal or diffuse GGOs and/or consolidation without specific distribution. "Crazy paving sign".
3. **Atypical:** Uncommon or not reported findings and recommend consideration of alternative diagnosis. Unilateral segmental or lobar consolidation, central unilateral or bilateral GGOs/ consolidation, discrete small nodules or tree-in-bud opacities, lung cavitation, pleural effusion, and lymphadenopathy.
4. **Negative:** No CT findings suggestive of pneumonia (Note: Chest CT may be negative in early stage)

## ULTRASOUND IN CHILDREN

Lung ultrasound (LUS) is a useful imaging modality especially in children as it is radiation-free. It provides the bedside diagnosis of many pulmonary conditions and status of the lung in an emergency as well as pediatric intensive care units (PICU). It is highly reliable for pneumonia, pleural effusion, and pneumothorax. Because of the peripheral or subpleural predominance of COVID-19, LUS can play a vital role in the early detection of the disease. An easier process of decontamination after the procedure is an added advantage in contrast to chest CT. Transportation of critically ill children can be difficult at times which can be solved through bedside ultrasound. However, the inability to detect the central consolidation and user-dependent are some of its limitations.^21-26^

A High-frequency transducer (15 MHz) is used and the intercostal spaces of the upper and lower parts of the anterior, lateral, and posterior regions of the bilateral chest are examined with a total of 12 regions. Four patterns were defined and given a score for each: 1. Normal aeration: the presence of lung sliding and artifactual horizontal A-lines (0 points), 2. Loss of lung aeration resulting from the scattered foci of bronchopneumonia or interstitial syndrome: the presence of multiple well-defined vertical B-lines extending from the pleural line or a small subpleural consolidation (1 point), 3. Loss of lung aeration resulting from alveolar-interstitial edema that corresponds to the CT imaging entity of the ground-glass: multiple confluent vertical B-lines extending from the pleural line or a small subpleural consolidation (2 points), 4. Lung consolidation characterizing extensive bronchopneumonia: the presence of a tissue structure containing hyper-echoic punctate foci representative of air bronchogram (3 points). A global LUS aeration score ranging from 0 to 36 was obtained by summing the individual scores of all the regions. The high US score was seen in moderate to severe disease which is consistent with CT findings.^21,22^

In a study done by Denina, et al,^22^ LUS performed in 8 patients, paying particular attention to signs of viral pneumonia as small subpleural consolidations and/ or individual B-lines or confluent B-lines (echogenic vertical lines arising from the pleural line and moving in concert with a sliding lung, expression of an interstitial syndrome). LUS revealed subpleural consolidations in 2 children and confluent B-lines in 5 children. In 7 of 8 patients, we found a concordance with the radiologic findings, whereas in the remaining patient, an interstitial B-lines pattern was observed despite normal chest radiography. One patient with severe clinical type was repeatedly examined with LUS on alternate days, and we noted a B-lines bilateral pattern reduction a day in advance before clinical and radiographic improvement. However, large-scale studies are required for its validation and need to expand evidence in point-of-care ultrasound in children.

## SPECTRUM OF IMAGING FINDINGS IN CHEST

In a retrospective literature review of 39 published articles involving 850 pediatric populations (<18 years and COVID-19 RT-PCR positive), 26.5% of patients had normal CT findings and 55% had involvement of unilateral lung fields. Ground-glass opacities and consolidations were the most common CT abnormalities (61.5%). A combination of GGOs and consolidation was found in 3.7% and other findings were halo sign, interstitial opacities, bronchial wall thickening, and crazy-paving sign.^27^

Another study done in Spain showed that CXRs were performed in 35 patients (80 % of admissions) and the most common indications were fever and respiratory symptoms. 53 % of the chest X-rays were considered normal and the classical bilateral diffuse interstitial pattern, described in adults, was only present in 22 %. All patients with pathological chest X-rays were symptomatic and reported fever (100 %) and fever tended to be longer (fever duration: 4.25 vs. 2.46 days p: 0.048) in patients with pathological radiographs. We present a specific protocol for chest imaging in pediatric COVID-19 cases.^28^

Bronchial wall thickening is a common finding in children. In RT-PCR positive cases of COVID-19, Chen et. al identified ground-glass opacities were present in 42.9%, bronchial wall thickening in 28.6%, and GGOs as well as consolidations along with nodular opacities in 14.3% of cases.^29^ After reviewing the imaging findings in RT-PCR positive patients (age 10 months to 18 years) from the six centers in China, CT findings were negative in 77% of the patients. Crazy paving patterns were observed in 29% of the patients and 23% had GGOs and peripheral lung consolidation. Predominant patterns of 86% of the cases were peripheral or sub-pleural. However, pleural effusion, lymphadenopathy, pulmonary nodularity, and fibrosis are absent in all cases. There was a positive correlation between increasing age and severity of the clinical and radiological manifestations.^30^ Yu H, et al. performed CT on 82 RT-PCR positive children and reported unilateral pneumonia in 46% and bilateral distribution were present in 36.5% of cases.^31^ Bilateral and multifocal involvement were seen in 55% of CT examinations and single lesion and single lobe involvement were detected in 27% of cases. Pure ground glass appearance was observed in 41%, ground-glass appearance and consolidation together were seen in 36% of the cases. There was significant involvement of the lower lobes and peripheral and central co-distribution of the lesions were frequently observed. There were coexistence of multiple rounded multifocal ground-glass appearance and rounded consolidation.^32^

In a study performed in neonates to 16 years children with confirmed COVID-19 in a tertiary pediatric hospital in Spain, fever was the most common symptom (43.5%). 90% of CXRs showed abnormalities. Peribronchial cuffing was the most common finding (86.3%) followed by GGOs (50%). In the majority of cases, central distribution was more common than peripheral. Follow-up CXRs were also performed in children depending on clinical evolution. Most of the children (75%) were completely recovered and underwent CXRs on discharge which showed complete resolution. Unfavorable outcomes were seen in the children having bilateral diffuse opacities with peribronchial cuffing and GGOs.^33^ CT findings were abnormal in 17 (71%) patients, with 5 (21%), 9 (38%), and 3 (13%) patients considered to have typical, indeterminate, and atypical findings, respectively. The most common CT patterns were multiple ground-glass opacities (58%), followed by consolidations (50%). Six patients showed the predominantly peripheral distribution of parenchymal abnormalities. A halo sign was identified in 3 patients and a perilobular pattern was identified in one of the cases with typical findings.^34^

## DIFFERENCES FROM ADULTS

Most of the children have normal imaging similar to adults. However, there are few points predominantly observed in children which are not commonly seen in the adult population. Clinical signs and symptoms are often lesser and milder in children than in the adult population. More focal abnormalities are seen in children, typically as GGOs and consolidations with unilateral lower lobe predominance. Several lobe's involvements are lesser in children. Peribronchial cuffing or bronchial wall involvement was observed in children. Multi-systemic involvement is also one of the particular manifestations of COVID-19 in children in contrast to adults.^29^

## BEYOND PNEUMONIA

In recent times, a new syndrome associated with severe COVID infection was coined which is known as pediatric multisystem inflammatory syndrome (PMIS) or multisystem inflammatory syndrome in children (MIS-C). It is one of the peculiar characteristics in children with COVID-19.^35-39^ MIS-C associated with COVID-19 is characterized by persistent fever associated with abdominal pain, vomiting, diarrhea, rash, conjunctivitis, and other mucocutaneous manifestations.^36-44^

Case definitions for post-COVID-19 hyperinflammatory syndrome according to World Health Organization (WHO)^35^ are children and adolescents (0-19 years) with fever for ≥3 days and two of the following symptoms: 1. Rash or bilateral non-purulent conjunctivitis or mucocutaneous inflammation signs (oral, hand, or feet), 2. Hypotension/shock, 3. Features suggestive of myocardial dysfunction, pericarditis, valvulitis, or coronary abnormalities (including echocardiographic findings or elevated troponin/NT-pro BNP), 4. Evidence of coagulopathy (using PT, PTT, elevated D-dimer), 5. Acute gastrointestinal symptoms (diarrhea, abdominal pain, or vomiting) and elevated markers of inflammation (CRP, ESR, or procalcitonin) and no other obvious microbial cause of inflammation, including bacterial sepsis, staphylococcal, or streptococcal shock syndromes and evidence of COVID-19 (RT-PCR assay, antigen test, or serology positive) or likely contact with patients with COVID-19. This syndrome should be considered in children with features of typical or atypical Kawasaki disease or toxic shock syndrome.

Similarly, case definitions given by CDC^35^ include 1. An individual aged <21 years presenting with fever, laboratory evidence of inflammation, and evidence of clinically severe illness requiring hospitalization with multisystem (>2) organ involvement (cardiac, renal, respiratory, hematologic, gastrointestinal, dermatologic, or neurologic), 2. Fever >38.0°C for ≥24 hours or report of subjective fever ≥24 hours, 3. At least one of the following laboratory findings: an elevated CRP level, ESR, fibrinogen, procalcitonin, d-dimer, ferritin, lactic acid dehydrogenase, or IL-6 levels; elevated neutrophil level; reduced lymphocyte level; and low albumin level, 4. No alternative plausible diagnosis and 5. Positive for current or recent SARS-CoV-2 infection at RT-PCR assay, serology, or antigen test or COVID-19 exposure within the 4 weeks before the onset of symptoms, or 6. Additional comments state that some individuals may fulfill full or partial criteria for Kawasaki disease but should be reported if they meet the case definition for MIS-C. MIS-C should be considered in any pediatric death with evidence of SARS-CoV-2 infection.^35^

There was a range of multisystem abnormalities in children. The abnormalities were 1. Cardiovascular (51%): cardiomegaly, pericardial effusion, pancarditis, myocarditis, decreased myocardial contractility, coronary artery aneurysms (20%), 2. Gastrointestinal/ hepatobiliary: Ascites, mesenteric inflammatory change, right iliac fossa lymphadenopathy (37%), bowel wall thickening in right iliac fossa (29%), splenic infarcts, gallbladder sludge, and wall thickening, 3. Respiratory: perihilar bronchial wall/interstitial thickening (34%), atelectasis (often fleeting), consolidation: perihilar or lower lobes, round pulmonary consolidations with ground-glass halo, pleural effusions (11%), 4. Central nervous system: large hemispheric infarct (one case; most likely secondary to extracorporeal membrane oxygenation).^25^ In children with MIS-C associated with COVID-19, the most common thoracic imaging abnormalities were cardiomegaly, congestive heart failure, or pulmonary edema and effusions. Interestingly, in patients with COVID-19, consolidations/ GGOs were the most common abnormality. Common abdominal abnormalities were ascites, hepatomegaly, and echogenic kidneys (present in 1/3^rd^ of the patients due to multi-organ injury).^36^

Children with MIS-C were more likely to have interstitial opacities and pleural effusions.^37^ The study done by EP Fenlon et. al identified that 82% of chest radiographs in children infected with COVID-19 had some findings. Pulmonary opacities were the most common findings in children (62%), which were bilateral and diffuse.

Bronchial wall thickening was present in 58%. On abdominal findings, minimal ascites was present in 54% of cases and other findings were bowel wall thickening in the right iliac fossa along with lymphadenopathy.^38^

Neurological manifestations in children with COVID-19 were identified by the ASPNR PECOBIG collaborator group. The most common imaging findings were posted infectious immune-mediated acute disseminated encephalomyelitis-like changes of the brain, myelitis, and neural enhancement. Patients had splenial lesions and had myositis in children with MIS-C. Cerebrovascular complications in children were less common than in adults. Significant preexisting comorbidities were absent and most children had favorable outcomes.^39,45^

Another interesting aspect of the COVID-19 era is highlighted by Bottari G et. al that 71% decrease in imaging studies and the proportion of negative imaging studies (with no evidence of bone fractures) dropped in 2020 by 19% compared to the 2019 control era. The sharp decrease of negative studies suggests that the rate of appropriateness was higher during COVID-era, suggesting some attitude toward defensive medicine in the previous control year, as a result of some degree of imaging inappropriateness.^46^

Radiographic features of MIS-C included pleural effusions (82%), pulmonary consolidations (73%), and ground-glass opacities (91%). All of the lung opacities (100%) were bilateral, and the majority of the pleural effusions (67%) were bilateral. Compared to children with COVID-19, children with MIS-C were significantly more likely to develop pleural effusions on chest radiograph (82%) and a lower zone predominance of pulmonary opacifications (100% vs. 38%). Children with MIS-C who also had abdominal imaging had intra-abdominal inflammatory changes.^47^

## IMPACT ON PEDIATRIC RADIOLOGY DEPARTMENT AND STRATEGIES TO OVERCOME THE CHALLENGES

Based on the survey of the World Federation of Pediatric Imaging (WFPI) COVID-19 task force, the survey (response rate 87%) comprised representatives from 71 countries and Hong Kong across six continents. Sixty-six of 72 respondents (92%) indicated that COVID-19 has resulted in moderate (29%), significant (50%), or complete (13%) changes in radiology practices in their countries. The two most frequent concerns were personal/family health (75%) and exposure (67%). Seventy-nine percent of respondents indicated some level of discomfort in identifying pediatric COVID-19 imaging manifestations. Changes in resident education were reported by 94% of respondents, and 31% were concerned that the likelihood of current trainees pursuing a career in pediatric radiology will be impacted.^48^ A large reduction in overall outpatient imaging activity was shown by Langan et. al in a retrospective study. They showed that 49,250 records occurred in the 371 days post COVID-19 period compared with an expected 67,806 records pre COVID-19 period, representing 18,556 missed records.^49^

Imaging should be performed only when it is expected to alter patient management. To prevent disease transmission, it is important to manage the inpatient load effectively by triaging children, scheduling elective procedures, and managing symptomatic children and carers as COVID-19 positive until proven otherwise. Within the imaging departments, one should consider conducting portable examinations with COVID-19 machines or arranging dedicated imaging sessions. Finally, regular hygiene maintenance, usage of personal protective equipment, and regular disinfestations of imaging equipment should be done during the pandemic era.^50^

## IN SUMMARY

Evidence of manifestations of COVID-19 in children continues to evolve around the world. Imaging is not recommended for screening in children with mild symptoms. Multiple factors like the availability of RT-PCR tests, the sensitivity of early imaging in children should be taken into consideration. Radiological investigations must be done with caution in children with low doses following the ALARA principle. Multi-systemic inflammatory syndrome in children is a unique manifestation. Most of the multisystemic manifestations are typical in children in contrast to the adult population. International consensus and structured reporting algorithms guide the general as well as pediatric radiologists for uniform reporting around the world for this novel disease. Thus, radiologists must be vigilant for its early detection. Proper history, clinical evaluation and close discussion with pediatric clinicians is the most crucial process in diagnosis and treatment in children.

## References

[1] World Health Organization. (2021). COVID-19 [Internet]..

[2] Hu B, Guo H, Zhou P, Shi ZL (2020). Characteristics of SARS-CoV-2 and COVID-19.. Nature Reviews Microbiology..

[3] World Health Organization. (2021). WHO Coronavirus (COVID-19) Dashboard [Internet].. World Health Organization.

[4] World Health Organization. (2020). WHO Director-General's opening remarks at the media briefing on COVID-19 - 11 March 2020 [Internet]..

[5] Bastola A, Sah R, Rodriguez-Morales AJ, Lal BK, Jha R, Ojha HC (2020). The first 2019 novel coronavirus case in Nepal.. The Lancet Infectious Diseases..

[6] Chan JF, Yuan S, Kok KH, To KK, Chu H, Yang J (2020). A familial cluster of pneumonia associated with the 2019 novel coronavirus indicating person-to-person transmission: a study of a family cluster.. Lancet.

[7] U.S. Census Bureau. Quick Facts United States [Internet] (2021). https://www.census.gov/quickfacts/fact/table/US/AGE295219#AGE295219externalicon.

[8] Centers for Disease Control and Prevention. (2021). Demographic Trends of COVID-19 cases and deaths in the US reported to CDC [Internet]..

[9] Sharma A, Chapagain R, Bista K, Bohara R, Chand B Epidemiological and Clinical Profile of COVID-19 in Nepali Children: An Initial Experience.. Journal of Nepal Paediatric Society.

[10] Dong Y, Mo X, Hu Y, Qi X, Jiang F, Jiang Z (2020). Epidemiological characteristics of 2143 pediatric patients with 2019 coronavirus disease in China.. Pediatrics.

[11] Wiersinga WJ, Rhodes A, Cheng AC, Peacock SJ, Prescott HC (2020). Pathophysiology, transmission, diagnosis, and treatment of coronavirus disease 2019 (COVID-19): a review.. Jama..

[12] Parasher A (2021). COVID-19: Current understanding of its pathophysiology, clinical presentation and treatment.. Postgraduate Medical Journal..

[13] Ouassou H, Kharchoufa L, Bouhrim M, Daoudi NE, Imtara H, Bencheikh N (2020). The pathogenesis of coronavirus disease 2019 (COVID-19): evaluation and prevention.. Journal of immunology research..

[14] Shekerdemian LS, Mahmood NR, Wolfe KK, Riggs BJ, Ross CE, Kiernan CA (2020). Characteristics and outcomes of children with coronavirus disease 2019 (COVID-19) infection admitted to US and Canadian pediatric intensive care units.. JAMA pediatrics..

[15] Pinninti SG, Pati S, Poole C, Latting M, Seleme MC, Yarbrough A (2021). Virological characteristics of hospitalized children with SARS-CoV-2 infection.. Pediatrics..

[16] Li J, Long X, Wang X, Fang F, Lv X, Zhang D (2021). Radiology indispensable for tracking COVID-19.. Diagnostic and interventional imaging..

[17] Cao Q, Chen YC, Chen CL, Chiu CH (2020). SARS-CoV-2 infection in children: Transmission dynamics and clinical characteristics.. Journal of the Formosan Medical Association..

[18] Denina M, Scolfaro C, Silvestro E, Pruccoli G, Mignone F, Zoppo M (2020). Lung ultrasound in children with COVID-19.. Pediatrics..

[19] Giorno EP, De Paulis M, Sameshima YT, Weerdenburg K, Savoia P, Nanbu DY (2020). Point-of-care lung ultrasound imaging in pediatric COVID-19.. The ultrasound journal..

[20] Alilio PM, Ebeling-Koning NE, Roth KR, Desai T (2020). Lung point-of-care (POCUS) ultrasound in a pediatric COVID-19 case.. Radiology case reports..

[21] Kennedy TM, Malia L, Dessie A, Kessler DO, Ng L, Chiang EL (2020). Lung Point-of-Care Ultrasound in Pediatric COVID-19: A Case Series.. Pediatric emergency care..

[22] Copetti R, Cattarossi L (2008). Ultrasound diagnosis of pneumonia in children.. La radiologia medica..

[23] Katal S, Johnston S, Johnston J, Gholamrezanezhad A (2020). Imaging findings of SARS-CoV-2 infection in pediatrics: A systematic review of coronavirus disease 2019 (COVID-19) in 850 patients.. Academic Radiology..

[24] de Munain AI, Veintemilla CJ, Aguirre MH, Sánchez NV, Ramos-Lacuey B, Urretavizcaya-Martínez M (2021). Chest radiograph in hospitalized children with COVID-19. A review of findings and indications.. European Journal of Radiology Open..

[25] Chen A, Huang JX, Liao Y, Liu Z, Chen D, Yang C (2020). Differences in clinical and imaging presentation of pediatric patients with COVID-19 in comparison with adults.. Radiology: cardiothoracic imaging.

[26] Steinberger S, Lin B, Bernheim A, Chung M, Gao Y, Xie Z (2020). Little BP. CT features of coronavirus disease (COVID-19) in 30 pediatric patients.. American Journal of Roentgenology..

[27] Yu H, Cai Q, Dai X (2020). The clinical and epidemiological features and hints of 82 confirmed COVID-19 pediatric cases aged 0-16 in Wuhan, China.. MedRxiv..

[28] Palabiyik F, Kokurcan SO, Hatipoglu N, Cebeci SO, Inci E (2020). Imaging of COVID-19 pneumonia in children.. Br J Radiol.

[29] Oterino Serrano C, Alonso E, Andrés M, Buitrago N, Pérez Vigara A, Parrón Pajares M (2020). Pediatric chest x-ray in covid-19 infection.. European Journal of Radiology..

[30] Prata RP, Forjaco A, Ruano CA, Dias JL, Fernandes L, Ferreira A (2021). COVID-19 in a pediatric cohort — retrospective review of chest computer tomography findings.. Egyptian Journal of Radiology and Nuclear Medicine..

[31] Hameed S, Elbaaly H, Reid CE, Santos RM, Shivamurthy V, Wong J (2021). Spectrum of Imaging Findings at Chest Radiography, US, CT, and MRI in Multisystem Inflammatory Syndrome in Children Associated with COVID-19.. Radiology..

[32] Blumfield E, Levin TL, Kurian J, Lee EY, Liszewski MC (2021). Imaging findings in multisystem inflammatory syndrome in children (MIS-C) associated with coronavirus disease (COVID-19).. American Journal of Roentgenology..

[33] Biko DM, Ramirez-Suarez KI, Barrera CA, Banerjee A, Matsubara D, Kaplan SL (2021). Imaging of children with COVID-19: experience from a tertiary children's hospital in the United States.. Pediatric radiology..

[34] EP FI, Chen S, Ruzal-Shapiro CB, Jaramillo D, Maddocks AB (2021). Extracardiac imaging findings in COVID-19-associated multisystem inflammatory syndrome in children.. Pediatric Radiology..

[35] Rostad BS, Shah JH, Rostad CA, Jaggi P, Richer EJ, Linam LE (2021). Chest radiograph features of multisystem inflammatory syndrome in children (MIS-C) compared to pediatric COVID-19.. Pediatric radiology..

[36] Centers for Disease Control and Prevention. (2020). Multisystem Inflammatory Syndrome in Children (MIS-C) Associated with Coronavirus Disease 2019 (COVID-19) [Internet]..

[37] Royal College of Paediatrics and Child Health (RCPCH). (2020). Guidance-COVID-19 paediatric multisystem inflammatory syndrome [Internet]..

[38] World Health Organization. (2020). Multisystem inflammatory syndrome in children and adolescents with COVID-19 [Internet]..

[39] Whittaker E, Bamford A, Kenny J, Kaforou M, Jones CE, Shah P (2020). Clinical Characteristics of 58 Children With a Pediatric Inflammatory Multisystem Syndrome Temporally Associated With SARS-CoV-2.. JAMA.

[40] Toubiana J, Poirault C, Corsia A, Bajolle F, Fourgeaud J, Angoulvant F (2020). Kawasaki-like multisystem inflammatory syndrome in children during the covid-19 pandemic in Paris, France: prospective observational study.. BMJ..

[41] Lindan CE, Mankad K, Ram D, Kociolek LK, Silvera VM, Boddaert N (2021). Neuroimaging manifestations in children with SARS-CoV-2 infection: a multinational, multicentre collaborative study.. Lancet Child Adolesc Health..

[42] Bottari G, Stellacci G, Ferorelli D, Dell'Erba A, Arico M, Benevento M (2021). Imaging Appropriateness in Pediatric Radiology during COVID-19 Pandemic: A Retrospective Comparison with No COVID-19 Period.. Children..

[43] Riphagen S, Gomez X, Gonzalez-Martinez C, Wilkinson N, Theocharis P (2020). Hyperinflammatory shock in children during COVID-19 pandemic.. Lancet..

[44] Foust AM, Johnston PR, Kasznia-Brown J, Chu WC, Garcia-Pena P, Daltro P (2020). Perceived Impact of COVID-19 on Pediatric Radiology Departments around the World: World Federation of Pediatric Imaging COVID-19 Task Force Survey Results from Six Continents.. Radiology: Cardiothoracic Imaging.

[45] Langan D, Shelmerdine S, Taylor A, Bryant WA, Booth J, Sebire NJ (2021). Impact of the COVID-19 pandemic on radiology appointments in a tertiary children's hospital: a retrospective study.. BMJ Paediatrics Open..

[46] Raissaki M, Shelmerdine SC, Damasio MB, Toso S, Kvist O, Lovrenski J (2020). Management strategies for children with COVID-19: ESPR practical recommendations.. Pediatr Radiol..

